# Peer Effects on Engagement and Disengagement: Differential Contributions From Friends, Popular Peers, and the Entire Class

**DOI:** 10.3389/fpsyg.2021.726815

**Published:** 2021-09-27

**Authors:** Nina Steenberghs, Jeroen Lavrijsen, Bart Soenens, Karine Verschueren

**Affiliations:** ^1^School Psychology and Development in Context, Faculty of Psychology and Educational Sciences, KU Leuven, Leuven, Belgium; ^2^Developmental, Personality, and Social Psychology, Faculty of Psychology and Educational Sciences, Ghent University, Ghent, Belgium

**Keywords:** school engagement, peer effects, friendship, classmates, popularity, high-ability, self-esteem

## Abstract

School engagement and disengagement are important predictors of school success that are grounded in the social context of the classroom. This study used multilevel analysis to examine the contributions of the descriptive norms of friends, popular students and classmates regarding engagement and disengagement to the development of Students’ own behavioral and emotional engagement and disengagement among Flemish 7th-graders (*N* = 3,409). Moderating effects of Students’ self-esteem and cognitive ability were examined. The results showed effects from friends’ and classmates’ (dis)engagement on all dimensions of (dis)engagement. Popular Students’ engagement only affected individual Student’s behavioral disengagement and emotional engagement. Self-esteem and high cognitive ability did not make students more or less susceptible to peer effects.

## Introduction

School engagement is a predictor of several indicators of school success, such as academic achievement and attainment (Fredricks et al., 2004). It is also a malleable construct that is reactive to contextual factors (Skinner et al., 2008). In addition to parents and teachers, peers are assumed to play a key role in shaping Students’ engagement (Zhang et al., 2019).

School engagement is a multifaceted concept that describes Students’ behavioral, emotional, and cognitive involvement displayed by students in school (Fredricks et al., 2004). Behavioral engagement refers to positive conduct, involvement in learning and academic tasks, and participation in school-related activities. Emotional engagement is defined as the affective reactions of students in the classroom and the degree to which they feel a sense of belongingness in the school and in the classroom. Cognitive engagement can refer to the motivation to learn or to the appropriate use of learning strategies and self-regulated learning (Fredricks et al., 2004). Improving Students’ school engagement has been proposed as a way to increase academic achievement and their ability to cope with challenges (Fredricks et al., 2004).

Disengagement does not merely reflect low levels of engagement but instead involves active withdrawal from learning activities and the experience of negative emotions in the school context (rather than just an absence of positive emotions or active involvement). Like engagement, disengagement has a behavioral, emotional, and cognitive component (Skinner et al., 2008). Students who are disengaged in the way they behave, feel, and think are likely to display low-quality motivation for studying or even to have no motivation at all (Skinner et al., 2008), they often show more antisocial behavior (Müller et al., 2016), and they are at risk for adjustment problems and drop out (Skinner et al., 2008).

School engagement and disengagement are both dynamic concepts that are construed in interaction with the social context (Fredricks et al., 2004). Whereas the role of relationships with parents and teachers has received a lot of attention in the engagement literature (e.g., Vasquez et al., 2016; Roorda et al., 2017), less attention has been paid to the impact of peers on academic engagement. Most of these studies examined how the quality of Students’ peer relationships relates to classroom engagement (Wentzel, 2009). Sociometric studies found that peer acceptance contributes to more behavioral and emotional engagement, whereas peer rejection is related to lower engagement or even disengagement (Weyns et al., 2018).

In addition to shaping engagement through acceptance or rejection, peers can also have a normative effect on engagement. In the school context, peers are an important source of behavioral information, as peers interact on a regular basis, providing plenty of opportunities for influencing each other’s behavior (Ryan, 2000; Wentzel, 2009). Particularly during adolescence, the relevance of the peer group increases as success among peers becomes more important (Brechwald and Prinstein, 2011) and peer networks become more complex (Rubin et al., 2015).

Research on peer norms has focused mainly on Students’ perceptions of their environment (Hamm et al., 2011; Kwon and Lease, 2014). However, Students’ perceptions do not always correspond to reality and may be biased to justify a Student’s own behavior (Borsari and Carey, 2003). This study focuses on descriptive norms in the peer group. Descriptive norms refer to the norms that arise from the behavior of the members of a particular group (Lapinski and Rimal, 2005). When certain behaviors occur regularly in a group, these behaviors become part of the ruling norms in the group (Lapinski and Rimal, 2005).

Peer norms originate from the interactions and shared experiences between peers through information exchange and modeling (Ryan, 2000). First, when students discuss class situations and behaviors with peers, they are presented with new ideas and perspectives, and they gain knowledge about the opinions of other students. For example, adolescents have been found to be more likely to engage in maladaptive and deviant behavior when they are aware that their peers would also engage in this behavior (Hoeben and Thomas, 2019). Second, norms can be communicated through modeling processes. Through observation, students acquire information about the behavior of their peers as well as the reaction of other students and teachers to this behavior. This information allows students to adapt their own behavior (Ryan, 2000). In particular in adolescence, students spend more time with peers, allowing for more opportunities for modeling (Wentzel, 2009).

Adolescence is also a period characterized by a stronger opposition to achievement and effort (Hamm et al., 2011), leading to a general decrease in engagement and an increase in disengagement as students progress through secondary school (Fredricks et al., 2004). These developmental mechanisms can be strengthened or weakened by the norms prevailing in the peer group. Disruptive peer behaviors as perceived by students have been found to predict Students’ development of behavioral disengagement in adolescence (Müller et al., 2016). This means that when students experienced more disruptive behavior by their peers, their increase in disengagement was higher than when they observed more compliant behavior (Nelson and Debacker, 2008; Müller et al., 2016). Similarly students whose peers show high levels of engagement have been found to show a smaller decrease in engagement throughout secondary school (Kindermann, 2008).

Although research has begun to demonstrate effects of peer norms on students’ (dis)engagement, this research shows several lacunae. First, extant studies typically did not differentiate between behavioral and emotional engagement. There are, however, reasons to assume that certain dimensions of (dis)engagement are more susceptible to peer effects than others (Lapinski and Rimal, 2005). According to norm theory individuals will rely more on normative information from the peer group when they believe their behavior will be noticed by others. Behavior that is enacted publicly also allows for more opportunities for observation, and students are less likely to engage in information exchange about more private behavior (Ryan, 2000; Lapinski and Rimal, 2005).

Behaviors like classroom participation or disruptive behavior are very visible to other students. It is thus expected that these kinds of behavior are more affected by the norms prevailing in the peer group (Müller et al., 2016). In line with this reasoning, it could be hypothesized that peers have a stronger effect on behavioral (dis)engagement than on emotional (dis)engagement, given that behavioral indicators are typically more visible to peers than emotional indicators.

A second lacuna of research on peer effects is the lack of comparison between different types of peer groups. When looking for behavioral guidance, students will probably focus on a selection of peers (Brechwald and Prinstein, 2011). Especially when students grow older, their social network expands and becomes more complex (Hamm et al., 2011). As such, it is important to distinguish between peer groups, such as classmates, friends, and popular students (Müller et al., 2016).

Classmates spend a lot of time together and the classroom is an important context for interaction (Müller et al., 2016). Studies with primary and middle school students revealed that when students perceive their classmates as involved in classroom activities, they show more engagement themselves (Nelson and Debacker, 2008). Similarly, the mean classroom level of aggressive and disruptive behavior has been found to predict individual Students’ levels of aggressive and disruptive behavior (Müller et al., 2016).

However, friends are more personally important to an individual student than other classmates or they have a more prominent role in the class group. These more prominent or important classmates may have a stronger effect on individual students (Ryan, 2000). Friends in particular, form the center of a Student’s peer network. Students associate themselves with their friends voluntarily and they choose to spend time with them. All this time spent together provides more possibilities for the observation of behavior. In addition, to avoid conflict and rejection by friends, students are more willing to change their behavior even if this goes against their beliefs (Müller et al., 2016). Research on influence processes between friends has shown that friends affect each other’s adaptive behaviors (e.g., performance and prosocial behavior) and maladaptive behaviors (e.g., rule breaking, smoking, and alcohol use) (Kwon and Lease, 2014). A study on Finnish 10th-graders asked students to nominate the peers they like to hang out with. The study revealed that, over time and for all three dimensions of engagement, students became more similar to the peers they nominated (Wang et al., 2018). Most research on influence dynamics between friends is limited to the dyadic relationship between two students and does not regard friends as a group (Brechwald and Prinstein, 2011).

Especially during the adolescent years, students see their social world as an extensive hierarchy, with the most popular students situated at the top of the social pyramid (Kwon and Lease, 2014). Students who are perceived as popular use power, social dominance strategies and prosocial behavior to maintain their status (Kwon and Lease, 2014). Students looking to increase their social status might look at popular students as role models and copy their behavior to advance their own social position. Studies on perceived popularity found the engagement of popular students to be predictive for the engagement of individual students (Zhang et al., 2019). Also for disruptive behavior, Students’ perceptions of the behavior of popular students was found to predict individual behavior (Müller et al., 2016).

Classmates, friends, and popular students all can be expected to affect student behavior through the descriptive norms they install. However, few studies have compared the impact of these different peer groups. Müller et al. (2016) did not examine (dis)engagement but studied the effects of friends, classmates, and popular students on individual Students’ disruptive and aggressive behavior. In their study, the students nominated the members of a peer group and nominated peers who had enacted a certain behavior. They then calculated for every individual student the proportion of members of a peer group that had performed the behavior in question, according to the individual student. They found that every peer group contributed to a similar degree to the behavior of the individual. Zhang et al. (2019) found engagement of peers nominated as popular or likable by the individual to be predictive of individual engagement. However, they did not differentiate between different dimensions of engagement. In general, none of these studies focused on the differential effects of three types of peer groups on different domains of engagement and disengagement simultaneously. Also, they did not examine the unique contribution of one type of peer group, controlling for the other. As such, the unique effects of different peer groups on specific aspects of (dis)engagement are still poorly understood. Such an understanding of contextual influences on (dis)engagement is essential in creating effective interventions targeting poor academic achievement (Wang et al., 2018).

Although peer effects are assumed to be relevant to all adolescents, some students may be more susceptible to such effects than others. In other words, individual characteristics can have a moderating effect on the associations between peer group norms and engagement (Cohen and Prinstein, 2006). In addition to examining the peer effects of different types of peers, we explored the potential impact of two characteristics with direct relevance to Students’ susceptibility to peer norms: self-esteem and cognitive ability.

Global self-esteem captures Students’ evaluation of their general functioning as a person (Harter, 2006). Self-esteem is constructed in interaction with the social environment. Parents, teachers, and peers form the lens through which adolescents look at themselves and evaluate their behavior (Harter, 2006). Possibly, students who suffer from low self-esteem are more likely to adjust their behavior to the values and standards set by their peers, in order to gain validation from those peers. Whereas many scholars investigated how peers have an impact on self-esteem (Rubin et al., 2006), it remains unclear whether Students’ self-esteem affects their susceptibility to peer norms.

High-ability students might be more susceptible to influence from peers than their classmates. Just like their peers, high-ability students have a need to be accepted by their peers and to experience a sense of belongingness. At the same time, their cognitive ability and interests might make them feel different from their peers (Coleman et al., 2015), resulting in greater concerns about peer acceptance (Lee et al., 2012). To increase their acceptance and to avoid social rejection, scholars have argued that high-ability students may adapt their behavior to the prevailing norms (Swiatek, 2001). Other studies, however, have shown that students with higher cognitive capabilities have a better understanding of their social environment, with this knowledge strengthening their social position (Neihart, 1999), implying no stronger need to adapt their behavior according to peer norms. However, no research to date directly examined these conflicting claims by examining the interplay between peer norms and cognitive ability in students’ (dis)engagement.

This study aimed to contribute to the literature on peers and (dis)engagement in two ways, that is, (a) by adopting a multidimensional approach to (dis)engagement, and (b) by directly comparing effects of three types of peer groups Specifically, we tested whether peers have differential effects on the development of individual Students’ behavioral and emotional engagement and disengagement based on their social status or based on their relationship with the individual. We did this by comparing the contribution of actual descriptive peer norms on (dis)engagement among the whole class, the friend group, and the group of popular students to the prediction of the development of individual (dis)engagement over the course of one school year. Consistent with previous studies (Hamm et al., 2011; Dijkstra and Gest, 2015; Sentse et al., 2015; Engels et al., 2020), the descriptive norms of a peer group were operationalized as the average level of (dis)engagement among the members of the peer group in question.

We used a 2 × 2-approach to the assessment of engagement, thereby including both behavioral and emotional engagement and disengagement as outcomes. Cognitive (dis)engagement was not included in this analysis because, as was mentioned in the second paragraph of this section, scholars have yet to reach a consensus on the conceptualization and assessment of cognitive engagement (Fredricks et al., 2004).

We expected all peer groups to have a significant impact on the prediction of individual Students’ engagement and disengagement. Based on the literature review, it was not possible to forward explicit hypotheses about the unique and relative predictive power of the different peer groups (Müller et al., 2016; Zhang et al., 2019). We did hypothesize that behavioral engagement and disengagement would be affected more strongly by peers than emotional engagement and disengagement, as the former manifestations of (dis)engagement are more visible.

As a secondary aim, we examined the potential moderating role of two personal characteristics: self-esteem and high-ability. We expected low self-esteem to strengthen the impact of peer norms, such that students with low self-esteem will be affected more by the descriptive norms in their peer groups. Because of concerns about social acceptance and rejection, we expected high-ability students to be more susceptible to peer effects. Because previous literature suggests that boys and girls score differently on engagement and disengagement we controlled for the effect of gender (Wang et al., 2018).

## Materials and Methods

### Participants

This study used data from the TALENT-study, a longitudinal study among 3,409 Flemish students from 166 classes in 27 schools. The study was approved by the Ethical Committee of (anonymized for peer-review). This study used data from the first two waves, conducted in the school year 2017–2018. Individual (dis)engagement at Wave 2 (Spring of Grade 7) was predicted by initial individual (dis)engagement and peer group (dis)engagement based on nominations at Wave 1 (Fall of Grade 7). Students in the sample had just made the transition from primary to secondary school. At Wave 1, they had been with their classmates for 2–3 months. Students were on average 12.48 years old at Wave 1 and 49.91% of the students in the sample were male. Students came from slightly more advantaged social backgrounds than the general student population, with 14.1% having a mother without secondary school degree (compared to 18.0% in the population) and 11.9% speaking another language than Dutch at home (compared to 16.9% in the population).

### Measures

#### Individual Engagement Measures

School engagement and disengagement were measured with a shortened version of a questionnaire by Skinner et al. (2008), which has demonstrated adequate validity and reliability (Skinner et al., 2008). Behavioral engagement was measured using five items assessing Students’ effort, attention, and persistence in classroom activities (e.g., “I try hard to do well in school,” α_wave__1_ = 0.76, α_wave__2_ = 0.80). Behavioral disengagement was assessed using five items that tapped into Students’ lack of effort and withdrawal from learning activities (e.g., “When I’m in class, I just act like I’m working,” α_wave__1_ = 0.65, α_wave__2_ = 0.74). The five items measuring emotional engagement tapped into emotions that indicate wellbeing in class and participation in learning activities (e.g., *“*When we work on something in class, I feel interested,” α_wave__1_ = 0.73, α_wave__2_ = 0.81). Emotional disengagement was measured using seven items that captured emotions indicating Students’ alienation and distress during learning activities (e.g., “When we work on something in class, I feel discouraged,” α_wave__1_ = 0.81, α_wave__2_ = 0.83). The four types of (dis)engagement were measured at the beginning and at the end of the school year. The items were rated on a 5-Likert-scale ranging from *strongly disagree* to *strongly agree*.

#### Average Engagement and Disengagement of Peer Groups

Three different types of peer groups were defined: the class group, the group of friends, and the group of popular students.

##### Class group

Engagement and disengagement of classmates was defined as the average engagement and disengagement score for all students in the classroom. The (dis)engagement score of the individual student was excluded from the average.

##### Friends

Students were asked to nominate peers whom they consider friends (“Who are your best friends in your class?”). They could only nominate students in the same classroom and the number of nominations was unlimited, as suggested in the literature (Terry, 2000). The average engagement and disengagement scores of the friends was determined by calculating the average score of all the students nominated by the individual. Accordingly, this measure varied from student to student.

##### Popular students

In the peer nomination procedure, students were also asked to nominate the popular students in their class (“Who is most popular in your class?”). Students could nominate an unlimited number of classmates they perceived to be popular. The amount of received nominations was determined for every participant. In each class, students scoring above the 90th percentile receiving the most nominations were considered as popular students. This way, in each class between 2 and 7 were identified as popular, with an average of 3.1 students. The average engagement and disengagement scores within this group of popular students were calculated.

#### Self-Esteem

To measure self-esteem, the Dutch version of the Rosenberg Self-Esteem Scale was used (Rosenberg, 1965; Van der Linden et al., 1983). Previous research demonstrated the reliability and validity of this scale (Van der Linden et al., 1983). Students scored five items on a 5-Likert scale ranging from *strongly disagree* to *strongly agree* (e.g., “On the whole, I am satisfied with myself,” α = 0.85).

#### Cognitive Ability

All students participated in a cognitive test (CoVat-CHC; Magez et al., 2015) in the month before the first student survey. This test measures fluid and crystallized intelligence and has demonstrated both content validity (Tierens, 2017) and criterion validity (Magez and Bos, 2016). IQ-scores were calculated by comparing the results to a representative norming sample. This resulted in a normed score with population mean 100 and standard deviation 15. Students with an IQ above 120 were identified as high-ability students. Students with an IQ below 120 served as the reference group.

#### Gender

Gender was coded with males as the reference category, coded as 0, and females being coded as 1.

### Statistical Analysis

Multilevel analysis was used to account for the nesting of students in classes. All variables, except for gender and high-ability, were standardized to a mean of 0 and a standard deviation of 1. The intraclass correlation (ICC) and design effects (DEFF) were computed for every construct, all ICCs were significant. The ICC was 0.04 for behavioral engagement (DEFF = 1.52), 0.05 for behavioral disengagement (DEFF = 1.65), 0.06 for emotional engagement, and 0.07 for emotional disengagement (DEFF = 1.91). This means that only 4–7% of differences in (dis)engagement between students could be situated at the class level. Differences between students’ (dis)engagement scores are primarily explained by differences between students. The design effect is usually not interpreted as it is the ICC corrected for the sample size.

For every type of (dis)engagement, six models were tested. Model 1–3 tested the contribution of the average score of (dis)engagement of each peer group separately to the development of the (dis)engagement of the individual. Gender differences and initial scores for engagement were taken into account. Model 1 tested the contribution of the classmates, Model 2 of the friend group and Model 3 of the popular students. In Model 4 the average scores of all three peer groups were entered simultaneously in one model. By controlling for the initial (dis)engagement scores, the added value of the peer group scores was measured. Model 5 and 6 each included a moderator for the different peer group effects. Model 5 included self-esteem and Model 6 included high-ability as a moderator. All models included a main effect of the moderator and interaction effects for all three peer groups.

### Sensitivity Analyses

To test the robustness of our results, we ran a sensitivity analysis in which we tested whether the moderation analyses for cognitive ability depended on the operationalization. In the main analysis, the group of high-ability students (IQ ≥ 120) was compared to a reference group with IQ-scores below 120. We additionally performed analyses in which a moderation effect was tested treating IQ as a continuous variable.

### Missing Values

Of the initial sample, 33 students transferred to another school between T1 and T2. There were 264 students who did not report on their engagement and disengagement at T1 or T2 and 139 students who did not nominate any friends leading to an analytical sample of 2,929 students. Within this analytic sample, the individual missingness of any item did not exceed 4.47%. Missing items were handled using listwise deletion.

## Results

### Descriptive Statistics

Bivariate correlations between all variables are presented in Table 1 along with descriptive statistics. At both waves, engagement scores were positively correlated to each other and negatively correlated to both disengagement measures. Disengagement measures were positively related to each other. These correlations were found within each peer group as well with engagement measures of a peer group being correlated positively to each other and negatively to disengagement measures and disengagement measures being correlated positively to each other. Correlations between engagement levels of different peer groups were moderate, indicating that these peer groups overlap to some extent but are still sufficiently distinct to be examined as unique effects on students’ individual (dis)engagement. Regarding the moderator variables, self-esteem showed weak to moderate positive correlations with behavioral and emotional engagement and weak to moderate negative correlations with behavioral and emotional disengagement. High-ability showed only a few significant correlations with the other variables and all correlations were lower than 0.13.

**TABLE 1:** Bivariate correlations and descriptive statistics of the variables.

Variable	1	2	3	4	5	6	7	8	9	10	11	12	13	14	15	16	17	18	19	20	21	22	23
M	0.50	3.21	3.82	0.12	3.94	2.01	3.61	1.94	3.94	2.02	3.61	1.94	3.95	2.01	3.62	1.92	3.83	2.11	3.58	2.01	3.80	2.12	3.43
SD	0.50	1.07	0.86	0.32	0.62	0.65	0.72	0.67	0.18	0.20	0.23	0.22	0.41	0.43	0.46	0.43	0.30	0.32	0.34	0.33	0.69	0.71	0.79
1. Gender	−																						
2. Self-esteem	−0.01	−																					
3. High-ability	−0.08*	0.09*	−																				
4. T1 behavioral engagement	0.09*	0.23*	0.01	−																			
5. T1 behavioral disengagement	−0.09*	−0.38*	−0.10*	−0.47*	−																		
6. T1 emotional engagement	0.05*	0.29*	0.04*	0.56*	−0.38*	−																	
7. T1 emotional disengagement	−0.15*	−0.39*	−0.03	−0.47*	0.61*	−0.55*	−																
8. Class behavioral engagement	0.07*	0.11*	0.08*	0.29*	−0.19*	0.22*	−0.22*	−															
9. Class behavioral. disengagement	−0.03	−0.17*	−0.13*	−0.19*	0.30*	−0.20*	0.26*	−0.63*	−														
10. Class emotional engagement	0.04*	0.13*	0.12*	0.20*	−0.19*	0.32*	−0.25*	0.69*	−0.63*	−													
11. Class emotional disengagement	−0.07*	−0.17*	−0.12*	−0.19*	0.23*	−0.24*	0.33*	−0.66*	0.77*	−0.74*	−												
12. Friends behavioral engagement	0.11*	0.05*	0.04*	0.18*	−0.17*	0.14*	−0.17*	0.47*	−0.31*	0.32*	−0.32*	−											
13. Friends behavioral disengagement	−0.11*	−0.09*	−0.09*	−0.17*	0.21*	−0.17*	0.19*	−0.31*	0.48*	−0.31*	0.37*	−0.55*	−										
14. Friends emotional engagement	0.07*	0.06*	0.06*	0.14*	−0.16*	0.15*	−0.18*	0.36*	−0.33*	0.52*	−0.38*	0.59*	−0.47*	−									
15. Friends emotional disengagement	−0.18*	−0.08*	−0.05*	−0.18*	0.19*	−0.18*	0.23*	−0.33*	0.40*	−0.38*	0.51*	−0.54*	0.66*	−0.62*	−								
16. Popular behavioral engagement	0.05*	0.03	0.05*	0.16*	−0.13*	0.13*	−0.13*	0.55*	−0.42*	0.40*	−0.41*	0.31*	−0.22*	0.22*	−0.24*	−							
17. Popular behavioral disengagement	−0.01	−0.08*	−0.09*	−0.08*	0.18*	−0.11*	0.14*	−0.28*	0.59*	−0.34*	0.42*	−0.16*	0.30*	−0.19*	0.24*	−0.53*	−						
18. Popular emotional engagement	0.04*	0.06*	0.03	0.11*	−0.13*	0.18*	−0.14*	0.38*	−0.42*	0.56*	−0.43*	0.21*	−0.23*	0.31*	−0.27*	0.58*	−0.48*	−					
19. Popular emotional disengagement	−0.04*	−0.05*	−0.08*	−0.10*	0.13*	−0.13*	0.17*	−0.34*	0.44*	−0.39*	0.52*	−0.19*	0.23*	−0.20*	0.30*	−0.54*	0.62*	−0.64*	−				
20. T2 behavioral engagement	0.12*	0.17*	0.01	0.57*	−0.39*	0.38*	−0.40*	0.20*	−0.15*	0.14*	−0.15*	0.16*	−0.14*	0.12*	−0.15*	0.13*	−0.10*	0.09*	−0.08*	−			
21. T2 behavioral disengagement	−0.12*	−0.31*	−0.08*	−0.41*	0.54*	−0.33*	0.47*	−0.19*	0.22*	−0.16*	0.18*	−0.17*	0.17*	−0.13*	0.16*	−0.12*	0.15*	−0.12*	0.11*	−0.59*	−		
22. T2 emotional engagement	0.08*	0.25*	0.03	0.39*	−0.33*	0.54*	−0.46*	0.20*	−0.19*	0.22*	−0.20*	0.13*	−0.14*	0.14*	−0.16*	0.12*	−0.13*	0.14*	−0.12*	0.62*	−0.48*	−	
23. T2 emotional disengagement	−0.15*	−0.32*	−0.03	−0.34*	0.44*	−0.38*	0.56*	−0.17*	0.22*	−0.18*	0.22*	−0.14*	0.18*	−0.14*	0.19*	−0.10*	0.14*	−0.12*	0.10*	−0.49*	0.67*	−0.61*	−

*p < 0.05.

At the end of the school year, students were on average less engaged and more disengaged than at the beginning of the year. This observation was confirmed by a paired samples *t*-test on the unstandardized scores [behavioral engagement: *t*(3,067) = 14.30, *p* < 0.001; behavioral disengagement: *t*(3,067) = −9.69, *p* < 0.001; emotional engagement: *t*(3,067) = 14.13, *p* < 0.001; emotional disengagement: *t*(3,067) = −14.54, *p* < 0.001].

After controlling for gender, students with higher scores at T1 scored significantly higher at T2 for all engagement measures across all tested models (behavioral engagement: *b* = 0.56, *p* < 0.001; behavioral disengagement: *b* = 0.52, *p* < 0.001; emotional engagement: *b* = 0.52, *p* < 0.001; emotional disengagement: *b* = 0.55, *p* < 0.001).

The effect of gender was significant for all outcomes: on average, girls reported higher behavioral and emotional engagement and lower behavioral and emotional disengagement (behavioral engagement: *b* = 0.07, *p < 0.001;* behavioral disengagement: *b* = −0.07, *p* < 0.001; emotional engagement: *b* = 0.05, *p* = 0.002, emotional disengagement: *b* = −0.07, *p* < 0.001).

### Behavioral Engagement

Table 2 shows that when initial scores and gender are considered, the average engagement of the class significantly predicted the behavioral engagement of the individual at T2 according to Model 1 (*b* = 0.04, *p* = 0.031). Model 2 shows that the average engagement in the friends group also contributed to the prediction of behavioral engagement at T2 (*b* = 0.05, *p* < 0.001). The effect of the mean engagement of popular students in Model 3 was not significant (*b* = 0.03, *p* = 0.067). When all three peer group scores were included simultaneously, only the average engagement of friends (*b* = 0.05, *p* = 0.009) significantly predicted individual engagement; the effect of class average engagement was no longer significant.

**TABLE 2:** Multilevel models of peer group effects on behavioral engagement (*N* = 3,068).

Variable	Model 1 Class			Model 2 Friends			Model 3 Popular students			Model 4 All peer groups			Model 5 Self-esteem			Model 6 High-ability		
						
	*b*	*SE*	*t*	*b*	*SE*	*t*	*b*	*SE*	*t*	*b*	*SE*	*t*	*b*	*SE*	*t*	*b*	*SE*	*t*
Gender	0.07***	0.02	4.34	0.06***	0.02	3.88	0.07***	0.02	4.37	0.06***	0.02	3.87	0.06***	0.02	4.08	0.06***	0.02	3.93
Initial engagement	0.56***	0.02	35.96	0.56***	0.02	36.10	0.56***	0.02	36.91	0.55***	0.02	35.17	0.54***	0.02	33.64	0.55***	0.02	35.17
Self-esteem													0.05***	0.02	3.37			
High-ability																0.01	0.05	0.55
Class mean engagement	0.04*	0.02	2.16							0.02	0.02	0.70	0.02	0.02	0.64	0.02	0.02	0.66
Friends mean engagement				0.05***	0.02	3.33				0.05**	0.02	2.61	0.04*	0.02	2.49	0.04*	0.02	2.55
Popular students mean engagement							0.03	0.02	1.83	0.01	0.02	0.37	0.01	0.02	0.61	0.01	0.02	0.54
SeE * Class mean													−0.02	0.02	−1.12			
SeE * Friends mean													−0.01	0.02	−0.65			
SeE * Popular mean													0.02	0.02	1.23			
HA * Class mean																−0.01	0.02	−0.46
HA * Friends mean																−0.02	0.02	−1.05
HA * Popular mean																0.02	0.02	0.83
Variance at class level	0.02***	0.01		0.02**	0.02		0.02***	0.01		0.02**	0.01		0.02**	0.01		0.02**	0.01	
Residual variance	0.65***	0.02		0.64***	0.02		0.65***	0.02		0.64***	0.02		0.64***	0.02		0.54***	0.02	

*SeE, self-esteem; HA, high-ability.*

*p < 0.05 **p < 0.01, ***p < 0.001.

The addition of the interaction effects between peer group behavioral engagement and self-esteem (Model 5) or high-ability (Model 6) did not result in a significant interaction. The main effect of self-esteem on behavioral engagement in Model 5 was significant and positive (*b* = 0.05, *p* < 0.001).

### Behavioral Disengagement

Table 3 presents the results of the analyses for behavioral disengagement. When the peer groups were analyzed separately, in addition to gender and initial disengagement, all three peer groups contributed significantly to the prediction of an individual’s behavioral disengagement at T2 (*b_*class*_* = 0.07, *p* < 0.001; *b_*friends*_* = 0.05, *p* = 0.002; *b_*popular*_* = 0.05, *p* = 0.004). When all peer groups were included simultaneously (Model 4), only the effect of the classmates remained significant (*b* = 0.05, *p* = 0.034).

**TABLE 3:** Multilevel models of peer group effects on behavioral disengagement (*N* = 2,049).

Variable	Model 1 Class			Model 2 Friends			Model 3 Popular students			Model 4 All peer groups			Model 5 Self-esteem			Model 6 High-ability		
						
	*b*	*SE*	*t*	*b*	*SE*	*t*	*b*	*SE*	*t*	*b*	*SE*	*t*	*b*	*SE*	*t*	*b*	*SE*	*t*
Gender	−0.07***	0.02	−4.56	−0.06***	0.02	−4.08	−0.07***	0.02	−4.59	−0.07***	0.02	−4.2	−0.07***	0.02	−4.65	−0.07***	0.02	−4.38
Initial disengagement	0.52***	0.02	32.83	0.53***	0.02	33.05	0.53***	0.02	34.07	0.52***	0.02	31.70	0.47***	0.02	27.21	0.52***	0.02	31.46
Self-esteem													−0.14***	0.02	−8.18			
High-ability																−0.03	0.02	−1.85
Class mean disengagement	0.07***	0.02	3.69							0.05*	0.02	2.13	0.04	0.02	1.67	0.05*	0.02	2.02
Friends mean disengagement				0.05**	0.02	3.04				0.03	0.02	1.61	0.03	0.02	1.74	0.03	0.02	1.57
Popular students mean disengagement							0.05**	0.02	2.89	0.01	0.02	0.58	0.02	0.02	0.68	0.01	0.02	0.56
SeE * Class mean													0.003	0.02	0.13			
SeE * Friends mean													0.005	0.02	0.27			
SeE * Popular mean													−0.03	0.02	−1.30			
HA * Class mean																−0.02	0.02	−1.12
HA * Friends mean																0.01	0.02	0.77
HA * Popular mean																0.01	0.02	0.55
Variance at class level	0.01**	0.01		0.01**	0.01		0.01**	0.01		0.01*	0.01		0.02**	0.01		0.01*	0.01	
Residual variance	0.69***	0.02		0.69***	0.02		0.69***	0.02		0.69***	0.02		0.67***	0.02		0.69***	0.02	

*SeE, self-esteem; HA, high-ability.*

*p < 0.05, **p < 0.01, ***p < 0.001.

No significant interaction effects were found with self-esteem (Model 5) or high-ability (Model 6). The main effect of self-esteem was significant (and negative) in Model 5 after accounting for initial disengagement and gender (*b* = −0.14, *p* < 0.001).

### Emotional Engagement

As shown in Table 4, after accounting for initial score and gender, the average emotional engagement score of classmates, friends and popular students contributed to the development of the individual engagement when analyzed separately in Model 1, Model 2, and Model 3 (*b_*class*_* = 0.05, *p* = 0.019; *b_*friends*_* = 0.04, *p* = 0.006; *b_*popular*_* = 0.05, *p* = 0.013). When all peer groups were analyzed in the same model (Model 4), none of the peer group effects were significant.

**TABLE 4:** Multilevel models of peer group effects on emotional engagement (*N* = 2,047).

Variable	Model 1 Class			Model 2 Friends			Model 3 Popular students			Model 4 All peer groups			Model 5 Self-esteem			Model 6 High-ability		
						
	*b*	*SE*	*t*	*b*	*SE*	*t*	*b*	*SE*	*t*	*b*	*SE*	*t*	*b*	*SE*	*t*	*b*	*SE*	*t*
Gender	0.05**	0.02	3.01	0.04*	0.02	2.49	0.05**	0.02	2.99	0.04*	0.02	2.48	0.04**	0.02	2.74	0.04*	0.02	2.45
Initial engagement	0.52***	0.02	33.13	0.53***	0.02	33.86	0.53***	0.02	34.14	0.52***	0.02	32.52	0.50***	0.02	29.8	0.52***	0.02	32.49
Self-esteem													0.09***	0.02	5.53			
High-ability																−0.001	0.02	−0.06
Class mean engagement	0.05*	0.02	2.34							0.01	0.03	0.46	0.01	0.03	0.29	0.01	0.03	0.43
Friends mean engagement				0.04**	0.02	2.73				0.03	0.02	1.9	0.03	0.02	1.88	0.03	0.02	1.85
Popular students mean engagement							0.05*	0.02	2.48	0.03	0.02	1.42	0.04	0.02	1.53	0.04	0.02	1.49
SeE * Class mean													−0.04	0.02	−1.81			
SeE * Friends mean													0.04	0.02	2.37			
SeE * Popular mean													0.00001	0.02	0.00			
HA * Class mean																−0.04	0.02	−1.64
HA * Friends mean																0.03	0.02	1.52
HA * Popular mean																0.04	0.02	1.77
Variance at class level	0.03***	0.01		0.03***	0.01		0.03***	0.01		0.03***	0.01		0.03***	0.01		0.03***	0.01	
Residual variance	0.68***	0.02		0.67***	0.02		0.68***	0.02		0.67***	0.02		0.66***	0.02		0.67***	0.02	

*SeE, self-esteem; HA, high-ability.*

*p < 0.05, **p < 0.01, ***p < 0.001.

Effects of peer group engagement were not moderated by self-esteem (Model 5) or high-ability (Model 6). Model 6 did show a significant and positive effect of self-esteem on emotional engagement (*b* = 0.09, *p* < 0.001).

### Emotional Disengagement

Results for emotional disengagement can be found in Table 5. When the peer groups were analyzed separately (in Model 1, Model 2, and Model 3), classmates’ and friends’ average disengagement score contributed to the development of emotional disengagement of the individual (*b*_*class*_ = 0.04, *p* = 0.049; *b_*friends*_* = 0.06, *p* < 0.001). The effect of friends remained significant when the peer group scores were combined in Model 4 (*b* = 0.05, *p* = 0.003). Classmates’ and popular Students’ emotional disengagement levels showed no significant contribution in Model 4.

**TABLE 5:** Multilevel models of peer group effects on emotional disengagement (*N* = 2,047).

Variable	Model 1 Class			Model 2 Friends			Model 3 Popular students			Model 4 All peer groups			Model 5 Self-esteem			Model 6 High-ability		
						
	*b*	*SE*	*t*	*b*	*SE*	*t*	*b*	*SE*	*t*	*b*	*SE*	*t*	*b*	*SE*	*t*	*b*	*SE*	*t*
Gender	−0.07***	0.02	−4.45	−0.05***	0.02	−3.47	−0.07***	0.02	−4.48	−0.05***	0.02	−3.49	−0.07***	0.02	−4.16	−0.06***	0.02	−3.56
Initial disengagement	0.55***	0.02	34.49	0.55***	0.02	35.16	0.55***	0.02	35.80	0.55***	0.02	34.22	0.51***	0.02	29.33	0.55***	0.02	34.20
Self-esteem													−0.12***	0.02	−7.45			
High-ability																−0.01	0.02	−0.67
Class mean disengagement	0.04*	0.02	1.97							0.02	0.02	0.70	0.01	0.02	0.35	0.02	0.02	0.61
Friends mean disengagement				0.06***	0.02	3.42				0.05**	0.02	2.97	0.06**	0.02	3.16	0.05**	0.02	2.95
Popular students mean disengagement							0.01	0.02	0.56	−0.02	0.02	−0.72	−0.01	0.02	−0.59	−0.02	0.02	−0.67
SeE * Class mean													0.01	0.02	0.27			
SeE * Friends mean													0.03	0.02	1.76			
SeE * Popular mean													−0.03	0.02	−1.88			
HA * Class mean																−0.01	0.02	−0.60
HA * Friends mean																0.01	0.02	0.36
HA * Popular mean																0.02	0.02	0.90
Variance at class level	0.02***	0.01		0.02***	0.01		0.03***	0.01		0.03***	0.01		0.03***	0.01		0.03***	0.01	
Residual variance	0.66***	0.02		0.64***	0.02		0.66***	0.02		0.64***	0.02		0.63***	0.02		0.64***	0.02	

*SeE, self-esteem; HA, high-ability.*

*p < 0.05, **p < 0.01, ***p < 0.001.

The addition of self-esteem and high-ability as moderators in Model 5 and Model 6 did not result in any significant interaction effects. Findings of Model 5 showed that self-esteem significantly predicted decreases in emotional disengagement (*b* = −0.12, *p* < 0.001).

### Sensitivity Analysis

**FIGURE 1:**
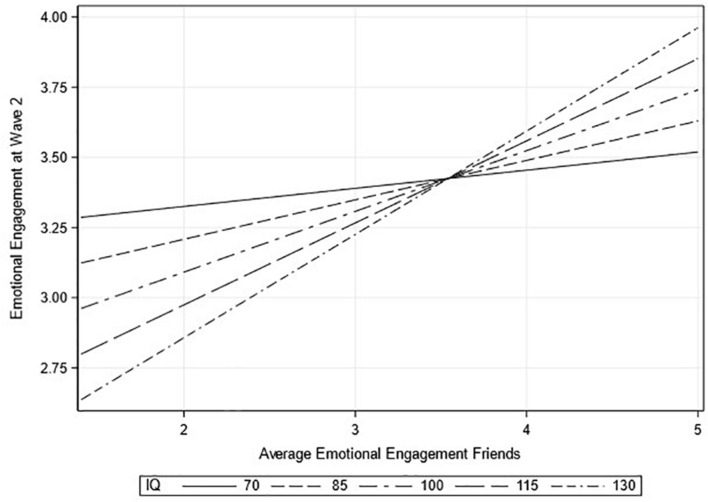
Interaction effect between the average emotional engagement of friends and IQ on emotional engagement.

To check whether the moderation effects of cognitive ability depended on the operationalization, the analyses were repeated with IQ treated as a continuous variable. All main effects of the peer groups that were significant in the main analysis remained significant in the sensitivity analysis. Treating IQ as a continuous variable did result in a significant and negative effect of IQ on behavioral disengagement (*b* = −0.04, *p* = 0.018) as was found in the main analysis. Lastly, a significant interaction between IQ and the average emotional engagement of friends was found. A graphical representation of this interaction can be found in Figure 1. This finding indicates a stronger effect of the emotional engagement of friends for students with a higher IQ (*b* = 0.04, *p* = 0.026).

## Discussion

This study compared the contribution of the engagement and disengagement of the whole class, friends, and popular students to the development of individual behavioral and emotional engagement and disengagement during Grade 7. The goal of the study was to gain more insight in the contribution of peers’ (dis)engagement the development of an individual’s (dis)engagement. Overall, the results indicate that peers do indeed shape the development of (dis)engagement of individuals, thereby confirming but also expanding earlier literature on school engagement (Sage and Kindermann, 1999; Kindermann, 2008).

The main research question was whether different peer groups have a differential impact on individual students’ (dis)engagement levels. When considered separately, both friends and classmates contributed to the development of both types of engagement and disengagement. Popular students contributed to behavioral disengagement and emotional engagement but not to behavioral engagement and emotional disengagement.

The findings in this study confirm the importance of friends to adolescents’ adjustment (Rubin et al., 2009), as they were shown to affect both behavioral and emotional engagement and disengagement. In the case of behavioral engagement and emotional disengagement, friends were the only group to have a unique contribution to individual Students’ engagement. As students progress through adolescence, friends play an increasingly prominent role in their life. Probably adolescents also look more at these friends for behavioral guidance. Also, students spend more time together with their friends after school, allowing for more discussions about class situations and more information exchange. No interaction effects were found, suggesting that the (dis)engagement level of the friend group has an impact on all students, regardless of their personal characteristics.

When considered separately from the other peer group types, classmates were found to be important to the development of individual Students’ behavioral and emotional engagement and disengagement. Additionally, when zooming in on the unique contribution of the classmates, thereby accounting for the (dis)engagement of friends and popular students, the class-average behavioral disengagement significantly predicted individual Students’ behavioral disengagement. This means that in classes where behavioral disengagement is high at the beginning of the school year, students will report a larger increase in disengagement toward the end of the school year than in classes with low average initial disengagement. Students with high behavioral disengagement will probably receive remarks from teachers, making behavioral disengagement very visible in the classroom setting. The high visibility of behavioral disengagement in the classroom allows for more modeling opportunities by classmates, which could explain why only the classmates had a unique impact on Students’ behavioral disengagement.

Popular students contributed to the development of behavioral disengagement and emotional engagement, although no unique contribution of this peer group to the development of (dis)engagement was found. Earlier literature found the behavior of popular students to be contributing to the development of anti-social and disruptive behavior (Müller et al., 2016). This study confirms the effect of popular students on disruptive behavior as it is highly similar to behavioral disengagement and extends the effect to emotional engagement. However, these results need to be interpreted carefully and without making strong claims as no unique contribution of popular students to the development of (dis)engagement was found.

Earlier research also suggested that students considered friends as a strong influence on their own behavior, but when asked who they think has the most influence on the behavior of others, they pointed at the popular students (Kwon and Lease, 2014). It is possible that popular students, rather than on *descriptive* norms, weigh more on *injunctive* class norms, that is, what people believe that others will think of their behavior and what needs to be done to avoid social sanctions (Lapinski and Rimal, 2005). By affecting what students think that ought to be done to gain acceptance or avoid rejection, they might affect individual students in a way that was not investigated in this study.

It was hypothesized that behavioral engagement and disengagement would be more affected by peer effects than emotional engagement and disengagement given the higher behavioral privacy of the latter dimensions of (dis)engagement. Consistent with this expectation, results indicated that none of the peer groups had a unique impact on individual Students’ emotional engagement. However, in the case of emotional disengagement, friends did have a, unique, albeit small, contribution to the disengagement of the individual. This finding may possibly be explained by the higher occurrence of co-rumination between friends. Co-rumination refers to the excessive discussion of problems with others (Rose et al., 2007). Research on depressive symptoms in adolescents has shown that higher levels of co-rumination with friends could lead to more depressive symptoms (Rose et al., 2007; Bastin et al., 2015). A consistent focus on the details of problems and negative feelings may lead to an overestimation of the importance of problems and to the perception that problems are more difficult to resolve (Rose et al., 2007). This mechanism can also be at play in the case of emotional disengagement. Negative feelings toward learning and school might be shared repeatedly among friends and therefore influence their feelings. Overall, then, the assumption that behavioral indicators of (dis)engagement would be affected more strongly by peer groups than emotional indicators was confirmed only partially.

Finally, this study investigated if students with low self-esteem or high cognitive abilities would be more susceptible to peer effects, but our hypotheses were not confirmed. We found that students with higher levels of self-esteem report higher levels of behavioral and emotional engagement and lower levels of behavioral and emotional disengagement and high-ability students were shown to report lower levels of behavioral disengagement. However, while these student characteristics were thus associated with (dis)engagement, the effects of peers on the development of (dis)engagement were not dependent on self-esteem or cognitive ability. Apparently, peer effects operate in the same way for all students. Of course, it could be that there are other student characteristics affecting Students’ susceptibility to peer effects that were not addressed in this study. For example, students with a high focus on popularity, the degree to which students attach importance to popularity and being popular themselves (Swiatek, 2001), are more oriented toward external validation and might therefore be more aware of and affected by the behavior of peers (Nelson and Debacker, 2008).

### Strengths and Weaknesses

This study has several strengths such as the use of peer nominations and the use of a population-based sample with a large number of students and classes. By comparing three different peer groups, this study contributed to the literature on peer norms in the classroom. The inclusion of four measurements for engagement and disengagement and two student characteristics (self-esteem and cognitive ability) provided a nuanced perspective on the mechanisms behind engagement and disengagement.

Even so, a few limitations of the study should be mentioned. First, the students in the sample transitioned from primary school to secondary school in September. Nominations of friends and popular students in November might change throughout the school year. The use of multiple waves of data within a school year could enable researchers to account for friendship stability. Second, students become increasingly aware of how they are perceived by others and attach more importance to social status as they emerge into adolescence (Hamm et al., 2011). Whereas the sample in this study consisted of early adolescents, maybe different results will be obtained in a sample of students in middle or late adolescence. Third, as this study focused on the perspective of the students, self-reports were used to measure engagement and disengagement. Studies using other informants for these constructs could strengthen this study’s results, although comparisons with teacher reports of student engagement have resulted in significant positive correlations between the scores of both informants (Skinner and Belmont, 1993). Fourth, the analyses revealed significant but low effect sizes. While this is not a limitation of the study design, it is important to keep this in mind in the interpretation of the results. Engagement is a complex construct that arises from the interplay of many factors. In this study, peer effects were approached from the perspective of norm theory, but there are also other ways in which peers can have an impact on the engagement of the individual, these include peer acceptance, bullying, romantic relationships, etc. Lastly, the sample investigated in this study consisted of students in the academic track of secondary education. The parent questionnaire that was also part of the research project revealed that parents of participating students were rather highly educated. This makes results less generalizable to a more heterogenous student population or to more vulnerable student populations.

### Implications for Practice

This study highlighted the importance of looking at classrooms as social systems where not only teachers but also peers play an important role in shaping Students’ adjustment. Results of this study emphasize the need for educators to incorporate the perspective of peer effects when designing interventions but also in their day-to-day practice.

Whereas peer influence is usually regarded as a negative element in Students’ development, this study shows that peers can also impact individual students in a positive way. Specifically, the findings suggest that increasing Students’ access to friends with high levels of engagement is one way to increase individual student engagement. Teachers can have a crucial impact on the social relationships in the classroom (Farmer et al., 2011). By taking up their role as a *social architect* in the classroom, they can regulate friendship relationships in their class. Research has shown, for example, that acceptance among classmates can be improved through seating arrangements in class (i.e., increasing physical proximity) (van den Berg and Cillessen, 2015). Educators can use these findings to increase the exposure of at-risk students to highly engaged peers. However, they must be vigilant that in doing so they do not bring about the undesirable effect whereby the at-risk students lower the engagement of the highly engaged students.

Furthermore, results suggest that engagement and disengagement can also be tackled at the class level. Several instructional approaches have shown to improve Students’ engagement (Reschly et al., 2020). The findings in this study suggest that such approaches will not only have a direct effect on individual engagement levels but will also improve Students’ individual engagement by raising the class level engagement.

## Data Availability Statement

The original contributions presented in the study are included in the article/supplementary material, further inquiries can be directed to the corresponding author/s.

## Ethics Statement

The studies involving human participants were reviewed and approved by the KU Leuven SMEC. Prior to conducting the study, informed consent was obtained from students and their parents or their legal guardian.

## Author Contributions

JL was responsible for the data collection and processing. NS performed the analysis, drafted the manuscript, and designed the figures. JL and KV supervised the work. All authors contributed to the conceptualization of the research, discussed the results, and commented on the manuscript.

## Conflict of Interest

The authors declare that the research was conducted in the absence of any commercial or financial relationships that could be construed as a potential conflict of interest.

## Publisher’s Note

All claims expressed in this article are solely those of the authors and do not necessarily represent those of their affiliated organizations, or those of the publisher, the editors and the reviewers. Any product that may be evaluated in this article, or claim that may be made by its manufacturer, is not guaranteed or endorsed by the publisher.
